# Oral Microbiome Shifts From Caries-Free to Caries-Affected Status in 3-Year-Old Chinese Children: A Longitudinal Study

**DOI:** 10.3389/fmicb.2018.02009

**Published:** 2018-08-28

**Authors:** He Xu, Jing Tian, Wenjing Hao, Qian Zhang, Qiong Zhou, Weihua Shi, Man Qin, Xuesong He, Feng Chen

**Affiliations:** ^1^Department of Pediatric Dentistry, Peking University School and Hospital of Stomatology, Beijing, China; ^2^Central Laboratory, Peking University School and Hospital of Stomatology, Beijing, China; ^3^The Forsyth Institute, Cambridge, MA, United States

**Keywords:** caries, oral microbiome shift, 3-year-old children, longitudinal study, Illumina sequencing, 16S rRNA gene, inter-bacterial correlation, caries-onset prediction model

## Abstract

As one of the most prevalent human infectious diseases, dental caries results from dysbiosis of the oral microbiota driven by multiple factors. However, most of caries studies were cross-sectional and mainly focused on the differences in the oral microbiota between caries-free (CF) and caries-affected (CA) populations, while little is known about the dynamic shift in microbial composition, and particularly the change in species association pattern during disease transition. Here, we reported a longitudinal study of a 12-month follow-up of a cohort of 3-year-old children. Oral examinations and supragingival plaque collections were carried out at the beginning and every subsequent 6 months, for a total of three time points. All the children were CF at enrollment. Children who developed caries at 6-month follow-up but had not received any dental treatment until the end of the study were incorporated into the CA group. Children who remained CF at the end of the study were incorporated into the CF group. Using Illumina Miseq Sequencing of the 16S rRNA gene, we monitored the shift of supragingival microbiome during caries initiation and progression in children who developed caries over the 12-month study period. Intriguingly, principle coordinates analyses revealed two major shifting patterns in microbial structures during caries initiation and progression in CA group, but not in CF group. Dynamic co-occurring OTU network study showed that compared to CF group, there was significant increase in both number and intensity of correlations between microbial taxa, as well as the formation of tight clusters of specific bacteria in CA group. Furthermore, there were enhanced correlations, positive ones between CA-enriched taxa, and negative ones between CF-enriched and CA-enriched species within CA group. Our data suggested coordinated microbial interactions could be essential to caries pathogenesis. Most importantly, our study indicated that significant microbial shifts occur not only during caries development, but even in the sub-clinical state. Using supragingival microbiome profiles, we were able to construct a caries-onset prediction model with a prediction accuracy of 93.1%. Our study indicated that the microbial shifts prior to the onset of caries might potentially be used for the early diagnosis and prediction of caries.

## Introduction

Dental caries is a multifactorial disease that results from interactions among a susceptive host, caries-related bacteria, and cariogenic diets. The earlier hypotheses attributed the etiology of the dental caries to either the increased total amount of plaque microflora (Non-specific Plaque Hypothesis) (Theilade, 1986; Marsh, 2006) or the presence of distinct cariogenic bacteria, such as *Streptococcus mutans* and *Streptococcus sobrinus*, within the plaque (Specific Plaque Hypothesis) (Loesche, 1986; Marsh, 2006). In recent years, the emergence of culture-independent molecular approaches has greatly expanded the spectrum of caries-related bacteria and allowed a better understanding of caries etiology. Currently, the “Ecological Plaque Hypothesis,” which reconciles the key elements of the earlier two hypotheses, has been widely accepted; it proposes that caries results from a shift in the balance of resident microbiota driven by changes in the oral environment (Marsh, 1994; Yang et al., 2012).

Most studies so far have mainly focused on analyzing the differences in the oral microbiota between caries-affected (CA) and caries-free (CF) populations. These cross-sectional studies revealed the difference in the richness and composition of the microbiome between the caries and healthy state, as well as identified many specific caries-related and health-associated bacterial species in different age groups (Chalmers et al., 2015; Johansson et al., 2015). However, due to their non-longitudinal nature, varying, sometime even contradictory results were obtained by different research groups. For example, Li et al. (2005) reported reduced richness and complexity of the bacterial population in dental plaque in children suffering from severe caries when compared with their CF counterparts; while Yang et al. (2012) showed that the microbiome in CA individuals were significantly more diverse in community structure than in healthy controls.

Only few cohort studies were longitudinal and focused on the microbiome changes with host growth and caries development process. Lif Holgerson et al. (2015) studied oral biofilms and saliva samples of children from 3 months to 3 years of age, and found that several bacterial taxa within the oral biofilms of the 3 years olds were associated with hosts’ CF or CA status. Hao et al. (2015) revealed a strong relationship between reduced microbial richness and caries onset using PCR-based denaturing gradient gel electrophoresis (PCR-DGGE). However, much remains to be elucidated regarding the dynamic structural shifts of oral microbiota and particularly the change in species association pattern during disease transition in the same subjects.

To better understand the polymicrobial nature of dental caries, we conducted a longitudinal study to investigate the dynamic changes in the supragingival microbiome of a cohort of 3-year-old children, who either stayed CF or developed caries over the 12-month study period, using Illumina Miseq Sequencing of 16S rRNA gene. We hypothesized that there would be developmental transitions in the oral microbiome in the healthy subjects as well as pathogenic shifts of oral microbiome during caries initiation and progression. Furthermore, we wanted to evaluate if the supragingival microbial profiles could be used for caries prediction, which would allow early diagnosis and intervention.

## Materials and Methods

### Ethics Statement

The Ethics Committee of Peking University Health Science Center (IRB00001052-5132) approved the study design, protocol, and informed consent procedure. Written informed consents were received from parents or guardians of all the participants prior to enrollment.

### Study Design

A total of 230 3-year-old children from 6 urban kindergartens in Haidian District, Beijing, China, underwent routine oral examination in March 2011. Among them, 144 children were recruited and followed up for 12 months. The exclusion criteria were: refusal to participate; have decayed, missing, or filled teeth due to caries (dmft > 0); enamel hypoplasia; dentin hypoplasia; white spots; systemic disease; use of antibiotics or topical fluoride application within the previous 3 months. Oral examinations and questionnaire surveys of children’s oral behavioral habits were carried out at enrollment (baseline at 0 month), 6- and 12-month follow-up.

All the children included in this study shared the same food menu, and kept the same oral cleaning habits during the daytime when they were in the kindergartens. Uniform oral health instructions leaflets, which included instructions of teeth brushing and dental flossing, were given out to parents by the kindergarten teachers. To ensure these children’s participation, reminder notifications for oral examinations, together with the questionnaires about oral habits were sent to parents 1 week before every follow-up. During the follow-up, if a child were found to be suffering from caries, a notification would be given to his/her parents/guardians, recommending them to take the child to a dental hospital or clinic for treatment.

All the 144 children were CF at enrollment. By the 6-month follow-up, 86 children remained CF; 31 children had developed caries but didn’t receive any dental treatment. These children were retained in the study. Another 13 children had developed caries and received treatment; 14 children dropped out because of personal reasons. These children were no longer followed up. By the 12-month follow-up, 58 children remained caries free. Among the 31 caries-active children at 6-month follow-up, 10 children still had not received any dental treatment by the end of 12-month follow-up, and they were incorporated into the CA group (**Supplementary Figure S1**).

### Clinical Examination and Sample Collection

All the dental examinations and sample collections were conducted by one pediatric dentist and one assistant, using knee-to-knee technique, with an artificial light in the medical examination room of the kindergartens. Caries status and the decayed, missing, or filled teeth (dmft) index were scored according to the World Health Organization caries diagnostic criteria (WHO, 1997) with modification as follows. At baseline, children with any white spots on teeth were excluded from the study, while white spots were coded as caries during the follow-up appointment. No radiographs were taken. Consistency in examination and caries diagnosis was ensured by the provision of training for dental examiners prior to the initiation of the study. The kappa value for intra-examiner agreement in the diagnosis of caries was 0.834.

At each time point, full-mouth supragingival plaque samples from sound enamel surfaces were collected immediately after the oral examination (Li et al., 2006; Gross et al., 2010; Xu et al., 2014; Hao et al., 2015). All participants were instructed to refrain from cleaning their teeth for 12 h and to avoid food and drink for 2 h before sample collection. Plaque samples were collected between 9 and 11 a.m. The teeth were gently air-dried, and supragingival plaque samples were collected from all sound smooth surfaces using a sterile dental excavator and pooled into a sterile 1.5 ml centrifuge tube containing 50 μL TE (50 mM Tris-HCl, 1 mM EDTA; pH 8). Samples were immediately shipped on dry ice to the microbiology laboratory at Peking University School of Stomatology within 60 min. All samples were washed in 1 mL TE buffer twice, centrifuged, and the precipitates were stored at –80°C until use.

### Total Genomic DNA Extraction and Illumina Sequencing Analysis of 16S rRNA Gene Amplicons

Total genomic DNA of the plaque samples was extracted using a Wizard Genomic DNA Purification Kit (Promega, Madison, WI, United States) according to the “Isolating Genomic DNA from Gram Positive and Gram Negative Bacteria” protocol. The quantity and quality of the extracted DNA was evaluated using a NanoDrop 8,000 spectrophotometer (Thermo Fisher Scientific, Waltham, MA, United States). Based on the quantity and quality of isolated DNA, 19 out of the 58 CF children samples were selected for the 16S rRNA gene sequencing. They were incorporated into the CF group. V3–V4 hypervariable region of 16S rRNA gene was PCR amplified and then sequenced by an Illumina Miseq Sequencing platform (paired-end 300) at the BGI (Huada Gene Institute) (Tian et al., 2015). During the experiment process, DNA extractions were performed by the same two persons within a total of 5 days to minimize the risk of possible contaminations between samples or by the researchers. The sequencing procedure in BGI company was mainly performed by two persons and with another person to double check all the steps.

### Bioinformatics Analysis

The end 100 bp of read2 were cut off and the sequences were trimmed by a quality score of 30 to ensure high sequence quality. Based on the barcodes, the sequences were assigned to different samples; the barcodes and primers were trimmed off. Pair-end sequences were joined together using PANDAseq (Masella et al., 2012). Joined sequences were clustered into operational taxonomic units (OTUs) at the 3% dissimilarity level using UPARSE pipeline (Edgar, 2013), and taxonomies were assigned by using BLAST against GreenGenes Database using the RDP classifier, with a confidence threshold of 0.8. PICRUSt (phylogenetic investigation of communities by reconstruction of unobserved states) was used to predict functional profiling of microbial communities using 16S rRNA gene sequences obtained above (Langille et al., 2013).

Principal coordinate analysis (PCoA) was performed using QIIME (Caporaso et al., 2010). This analysis is a multivariate statistical technique for finding the most important dimensions along which samples vary. The principal components (PCs) describe the degree of variation each of the dimensions accounts for Lozupone and Knight (2005). Dissimilarity analysis of beta diversity was calculated using the adonis function. Co-occurrence networks were calculated using R software (with packages psych and reshape2; spearman test) and visualized with Cytoscape 3.2.1 (Shannon et al., 2003). Mean differences in the relative abundance of OTUs and predicted metabolic pathways between and within groups were evaluated using LEfSe (Segata et al., 2011), with an alpha value of 0.05 for the Kruskal–Wallis test and a threshold of 2.0 for logarithmic linear discriminant analysis scores. Comparison of alpha diversity was done by wilcoxon test for between-group calculation and Kruskal–Wallis test for within-group calculation using R software.

### Predictive Modeling of Caries Onset

One caries-onset prediction model was constructed using Random Forest methods with package Random Forest 4.6–10. The version of R software was 3.2.1. First, the relative abundances of OTUs were calculated using Wilcoxon Signed-Rank Test between the CA and CF groups at each time point, and 29 significantly different OTUs were detected. Corresponding vector of error rates (error.cv) were calculated by rfcv function among all the significantly different OTUs. Mean Decrease Accuracy and Mean Decrease Gini were calculated by importance function, and were applied to screen the most important OTUs (Breiman, 2001, 2002; Svetnik et al., 2004). Finally, 11 most important OTUs were screened out from these 29 OTUs. The formal prediction model was constructed based on the relative abundances of these 11 OTUs at the CA-baseline subgroup (CA-0m) and the CF-baseline subgroup (CF-0m). The power of this prediction model was preliminary validated using OTU relative abundances information of the 19 CF samples from the CF-6m subgroup.

## Results

### Participants

A total of twenty-nine subjects, ten in the CA group and nineteen from the CF group, were included in the final analysis and their supragingival samples were subjected to 16S rRNA gene sequencing. χ^2^ analysis and *t*-tests showed no significant differences between the two groups in terms of gender (*P* = 0.24) or age (*P* = 0.33). At each time point, both the children included in the final CA and CF group did not show significant differences in the frequency of eating sweets, frequency of eating sweets before sleep, drinking sugar-containing beverage or frequency of daily teeth brushing (**Supplementary Table S1**). In the CA group, the dt (the number of decayed teeth) value was 2.0 ± 1.1 at 6-month follow-up, and then significantly increased to 5.4 ± 3.0 at 12-month follow-up (*p* = 0.003) (**Supplementary Table S2**).

### General Information From Sequencing

A total of 803,769 high-quality reads were generated for the 29 samples in this study. On average, 9,239 reads per sample were obtained for analysis (**Supplementary Table S2**). Good’s coverage was ∼99.4–99.6% for all sequences in the six subgroups (CA-0m, CA-6m, CA-12m, CF-0m, CF-6m, CF-12m) (data not shown), indicating that only ∼0.5 additional phylotype would be detected for every 100 additional sequence reads. This coverage level indicated that these 16S rRNA gene sequences represented most of the phylotypes of plaque bacteria.

When clustering the unique sequences into OTUs at a 3% dissimilarity level, a total of 10 phyla, 16 classes, 21 orders, 38 families, and 55 genera were detected in these plaque samples. The distribution of bacterial relative abundances at phylum level within each sample was shown in **Supplementary Figure S2**. Six phyla (Proteobacteria, Fusobacteria, Firmicutes, Candidatus Saccharibacteria, Bacteroidetes, and Actinobacteria) were predominant in all groups, with other phyla presented in relatively low proportions.

Rarefaction curves were depicted in **Supplementary Figure S3**, microbial diversity within each sample (alpha diversity) was calculated based on a subsample of 2,773 reads from each dataset. The alpha diversity of the total bacterial microbiome in plaque was estimated using richness indices (Chao 1 and Observed OTUs) and diversity indices (Shannon and Simpson). Between-group and within-group calculation showed that there was no significant difference in the four indices of alpha diversity (**Supplementary Figure S4**).

### Shifts of Supragingival Microbiome Composition During Caries Development

**FIGURE 1:**
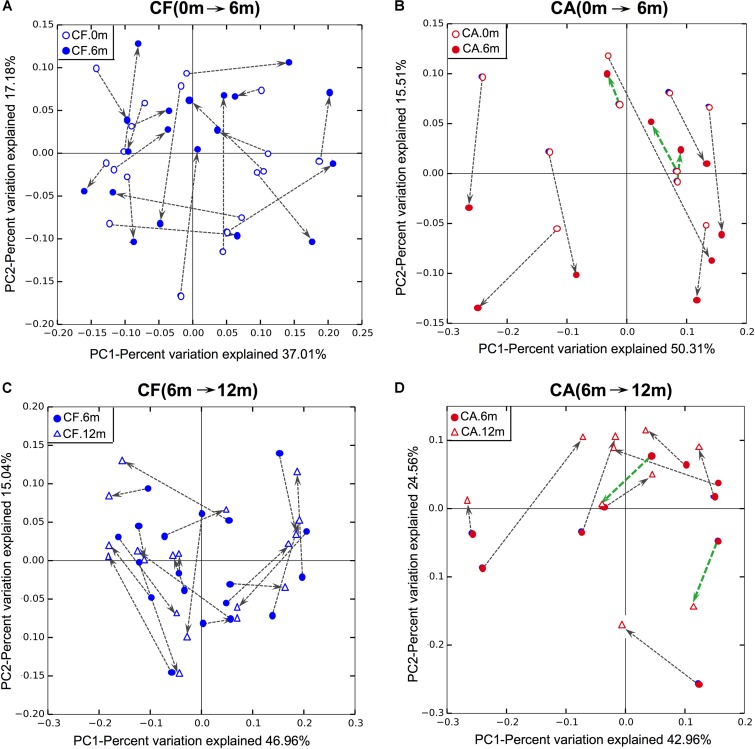
Principle coordinates analysis (PCoA) plot of supragingival microbiome community structure shift using weighted UniFrac distance. **(A,B)** Community structure shifts of CF and CA groups from baseline to 6-month follow-up. **(C,D)** Community structure shifts of CF and CA groups from 6- to 12-month follow-up.

In order to investigate shifts in the structure of the supragingival microbiome community during caries development and normal growth, principle coordinates analysis (PCoA) was conducted based on weighted unifrac distance (**Figure 1**). The results showed that, the microbiome in CA group displayed distinct shifting patterns during 12-month follow-up. From baseline to 6-month follow-up, the shifts in community structure mainly followed two major directions, with seven out of ten samples being shifted in one direction (gray arrows in **Figure 1B**), and the rest three shifted in another direction (green arrows in **Figure 1B**). Similarly, from 6- to 12-month follow-up, eight of the ten samples in the CA group shifted in one direction (gray arrows in **Figure 1D**), while the other two moved in another direction (green arrows in **Figure 1B**). On the contrary, no obvious shifting patterns were observed in CF group, either from baseline to 6-month, or from 6- to 12-month follow-up, as indicated by the arrows pointing to multiple directions (**Figures 1A,C**).

### Dynamic Co-occurring OTU Network of Supragingival Microbiome During Caries Development

**FIGURE 2:**
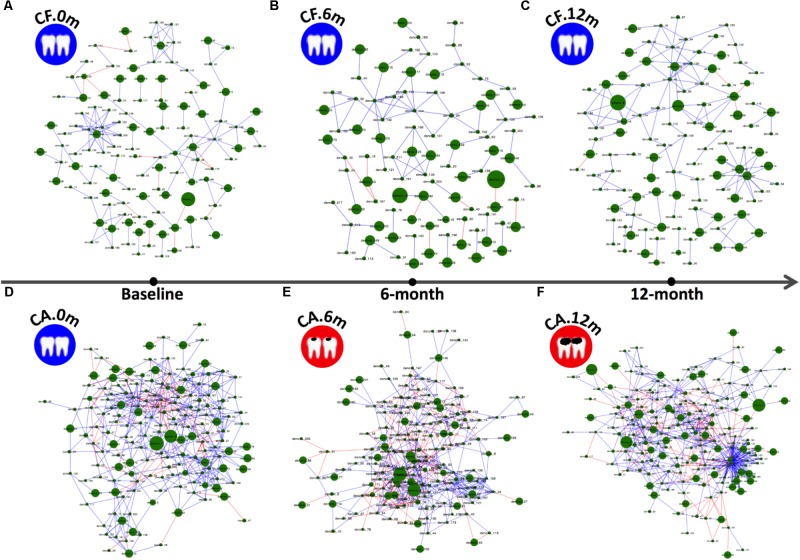
Co-occurring network and corresponding OTUs in supragingival microbiome of CF and CA groups during follow-up. Blue and red edges indicate positive (*r* > 0.4) and negative correlations (*r* < –0.4), respectively. The area of each node indicates the corresponding OTU’s relative abundance. **(A–C)** OTU co-occurring networks of supragingival microbiome of the CF group during follow-up. **(D–F)** OTU co-occurring networks of supragingival microbiome of the CA group during follow-up.

Bacterial species within a microbial community are often engaged in complex interspecies competition and/or cooperation. These dynamic interactions can be reflected by specific patterns of microbial associations as revealed by co-occurrence analysis. In an effort to investigate how the microbial correlation networks might change during follow-up, co-occurrence OTU networks of microbiome were analyzed (**Figure 2**), and the taxonomies of these OTUs were present in **Supplementary Data Sheet S1**. In both CA and CF groups, the co-occurrence networks shifted over time. However, the networks of the CA group displayed more drastic changes with more varied relative abundances of bacteria and more intense clusters formation. At each time point, including baseline when no caries can be detected for both groups, the CA group exhibited much more positive and negative correlations within OTUs, as well as formation of tighter bacterial clusters compared to CF group. Particularly, in the CA group at 12-month follow-up, OTUs that possessed the most connections with other OTUs mainly belonged to Lactobacillales [Dolosigranulum (OTU-179)] and Gammaproteobacteria [Pseudomonas (OTU-170), Acinetobacter (OTU-207), Enterobacteriaceae (OTU-85), and Cardiobacteriaceae (OTU-57)].

**FIGURE 3:**
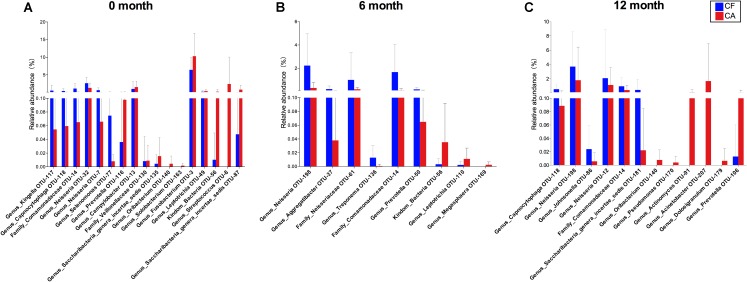
Comparison of OTU relative abundances in supragingival microbiome between CF and CA groups. Differences in relative abundance at OTU level were analyzed using the Wilcoxon rank-sum test, *P* < 0.05 was considered to reflect a significant difference. Not significant difference was not shown. The bars represent “mean ± SD” relative abundances. **(A)** Comparison of OTU relative abundances between CF and CA group at baseline. **(B)** Comparison of OTU relative abundances between CF and CA group at 6-month follow-up. **(C)** Comparison of OTU relative abundances between CF and CA group at 12-month follow-up.

**FIGURE 4:**
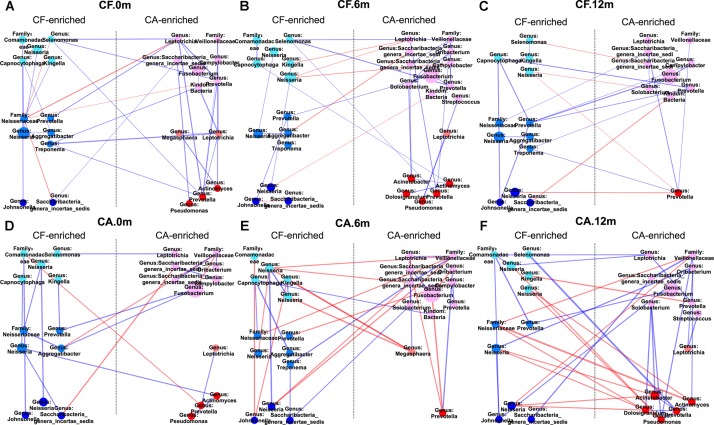
Co-occurring network of caries-free (CF) enriched and caries enriched OTUs. Blue and red edges indicate positive (*r* > 0.4) and negative correlations (*r* < –0.4), respectively. The area of each node indicates the corresponding OTU’s relative abundance. The width of each line that connects two dots indicates the closeness extent of the correlation. The three colors on the left side from top to bottom represent CF-0m-enriched, CF-6m-enriched and CF-12m-enriched OTU; the three colors on the right side from top to bottom represent CA-0m-enriched, CA-6m-enriched, CA-12m-enriched OTU. **(A–C)** Co-occurring networks of CF-enriched and CA-enriched OTUs in supragingival microbiome of the CF group during follow-up. **(D–F)** Co-occurring networks of CF-enriched and CA-enriched OTUs in supragingival microbiome of the CA group during follow-up.

Both PCoA analysis and the co-occurrence networks data suggested different microbiome compositions between CA and CF group at each time point. By comparing the relative bacterial abundances between the CA and CF groups, we were able to identify several CA-enriched and CF-enriched OTUs at each time point (**Figure 3**). CA-enriched OTUs included Streptococcus (OTU-8), Prevotella (OTU-116), Prevotella (OTU-50), Solobacterium (OTU-163) etc., while Kingella (OTU-117), Capnocytophaga (OTU-118), Neisseria (OTU-32), Fusobacterium (OTU-3) were among CF-enriched OTUs. To further investigate the potential interactions between these CA or CF-enriched taxa, co-occurrence networks were established based on their differential distribution and relative abundances within each subgroup at different time point (**Figure 4**). Correlations of these identified CA or CF-enriched taxa in the CA group displayed remarkable differences from the CF group even starting from baseline. In the CA group, both the number and intensity of the correlations among these CA-enriched and CF-enriched species increased during the follow-up. Particularly, at 12-month follow-up, four CA-enriched species [belonging to genera of Dolosigranulum (OTU-179), Pseudomonas (OTU-170), Acinetobacter (OTU-207), and Actinomyces (OTU-91)] displayed strong positive correlations with other CA-enriched species, such as Solobacterium (OTU-163) and Prevotella (OTU-106); as well as negative correlations with several CF-enriched species, such as Neisseria (OTU-198) and Neisseriaceae (OTU-61). Meanwhile, no strong correlations could be observed among CF- and CA-enriched bacterial taxa in CF group during the follow-up (**Figure 4**). These data further suggested the potential role of enhanced interactions among certain members within supragingival microbiome in the initiation and/or development of dental caries.

### Supragingival Microbiome Shift Before Caries Onset

**FIGURE 5:**
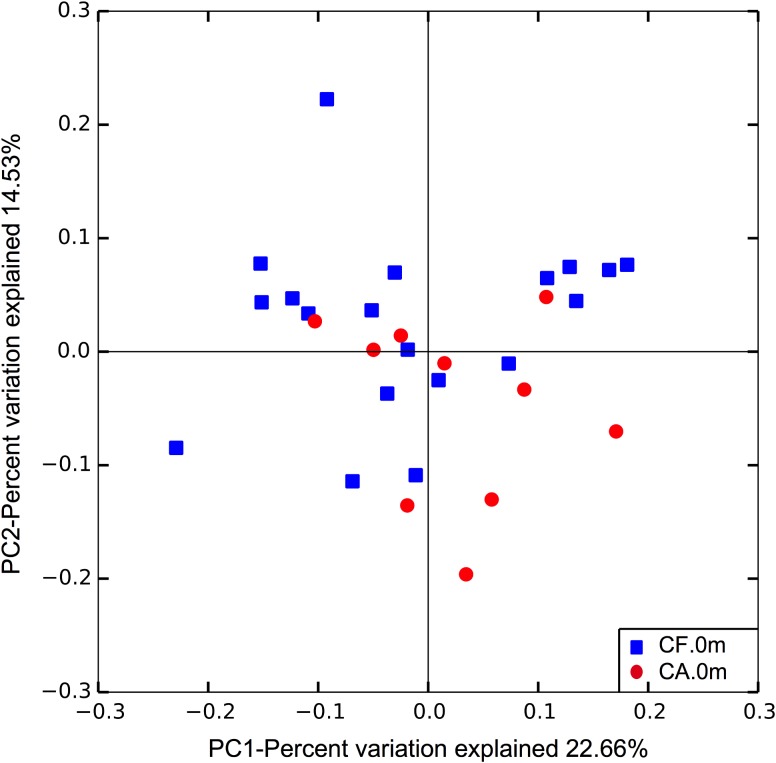
Principle coordinates analysis (PCoA) plot of supragingival microbiome community structure at baseline. The analysis was done based on unweighted UniFrac distance. Dissimilarity analysis done by Adonis exhibits *p* = 0.047.

To further compare the community structure of supragingival microbiome between CF and CA at baseline when no clinical manifestation of caries can be easily detected in both groups, the overall community composition was calculated based on the unweighted UniFrac distance and visualized with a PCoA plot. The analysis revealed distinct partitioning of microbial communities associated with CF or CA group (*p* = 0.047; **Figure 5**), suggesting that CF and CA groups carry distinct microbial communities prior to the onset of disease.

### Predictive Model of Caries Onset Based on Early Changes in the Supragingival Microbiome

**FIGURE 6:**
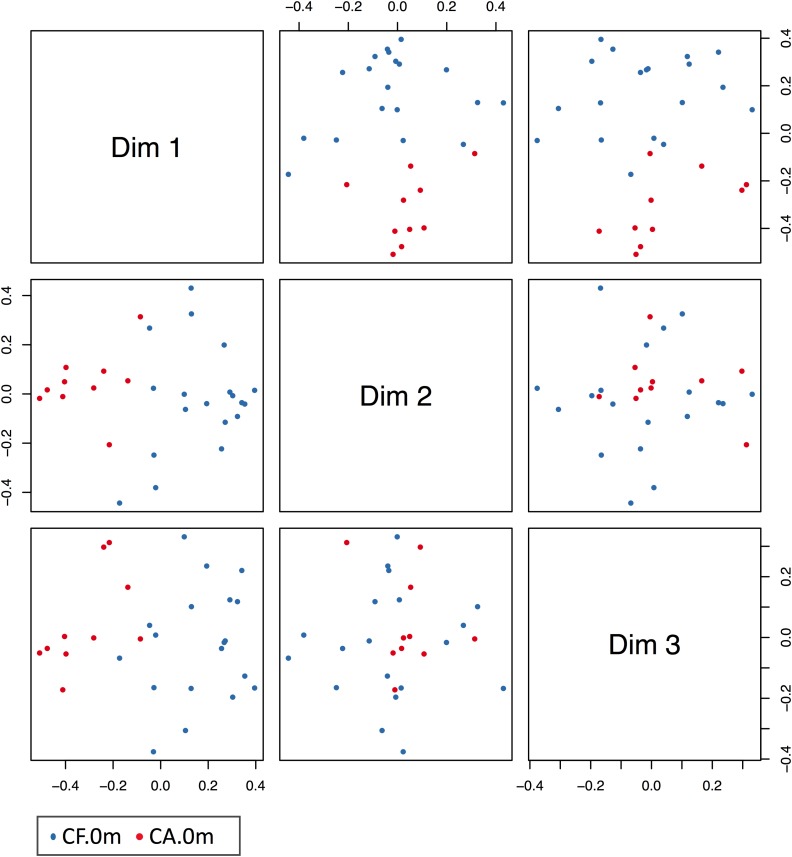
MSD figure of the prediction model of caries onset. Blue dots and red dots indicate CF and CA samples, respectively. In the figure, CF, and CA samples were largely separated at different dims’ level.

Based on our findings that the supragingival microbiome structural shifts could be monitored even before the onset of dental caries, we generated a random forest model for caries prediction. In this model, relative abundance information of the 11 most important OTUs in the CA-0m and CF-0m subgroups were included. The out-of-bag estimate of the error rate of this model was 6.9%, giving a caries prediction with accuracy of 93.1%, sensitivity of 83.3% (10/12), and specificity of 100% (17/17) (**Figure 6**). The ROC curve of prediction model was presented in **Supplementary Figure S5**, the AUC (Area Under Curve) of this ROC curve was 0.9474. We further confirmed the power of this model using CF-6m subgroups’ supragingival microbiomes, the validation accuracy was 89.47%. Prediction result of each sample was shown in **Supplementary Table S4**.

### Differential Carbohydrate Metabolism Capability Between Caries-Related and Health-Related Microbiome

**FIGURE 7:**
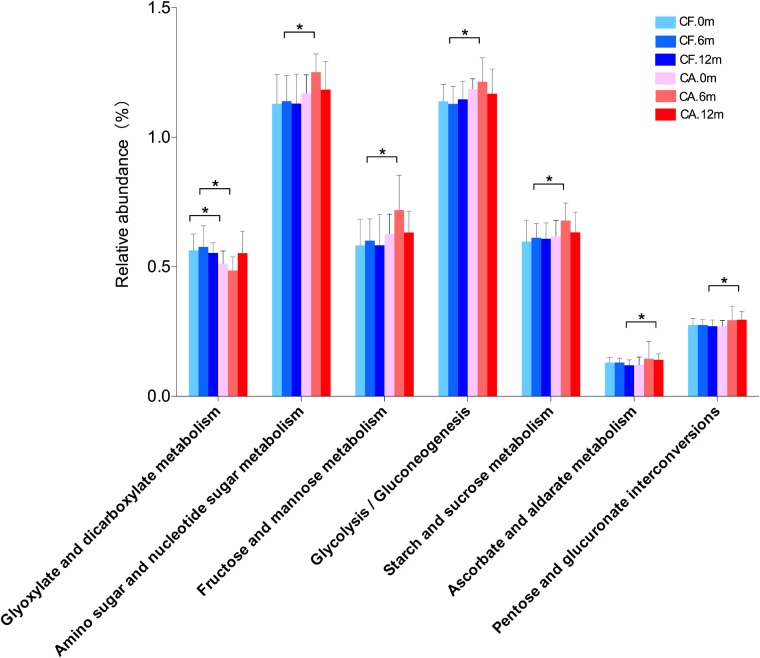
Comparison of carbohydrate metabolisms in supragingival microbiome between CF and CA groups. Differences in carbohydrate metabolism were analyzed using LEfSe analysis. The bars represent “mean ± SD” relative abundances. ^∗^*P* < 0.05.

We further performed preliminary microbiome metabolic pathways analysis in both CA and CF groups, particularly focusing on carbohydrate metabolism, since the sugar intake and metabolism play a significant role in caries initiation and development (Sheiham and James, 2015; Galvao-Moreira, 2016; James, 2016; **Supplementary Table S3**). Among the six carbohydrate related metabolisms, amino sugar and nucleotide sugar metabolism, fructose, and mannose metabolism, glycolysis/gluconeogenesis and starch and sucrose metabolism exhibited higher level in the CA group than the CF group at 6-month follow-up. In addition, ascorbate/aldarate metabolism and pentose/glucuronate interconversions exhibited higher level in the CA group than the CF group at 12-month follow-up. These up-regulated pathways in CA group suggest more active carbohydrate metabolisms, as well as more sugar-related bacteria activities during caries onset and development (**Figure 7**).

## Discussion

The current notion is that dental caries is a polymicrobial disease resulted from dysbiosis of oral microbiome. However, very little is known about the shift of community structure, particularly the reshaping of interspecies interactions during the transition in the same subjects. Furthermore, it remains an open question if/or how long a microbial shift can precede the clinical manifestation of dental caries. In this study, we monitored the microbial shift during caries initiation and progression by analyzing the dynamics of the supragingival microbiomes in children either staying CF or developing caries over the 12-month study period. Not only significant increase in both the number and intensity of correlations between microbial taxa, but also the formation of tight clusters of specific bacteria were observed during caries development. Furthermore, significant microbial shifts occur not only during caries development, but even in the sub-clinical state. One caries-onset prediction model was constructed based on these supragingival microbiome profiles. Our data suggested coordinated microbial interactions could be important in caries pathogenesis, and the microbial shifts prior to the onset of caries might potentially be used for the early diagnosis and prediction of caries.

The inclusion of 3- to 5-year-old children in our study was based on the literature that this particular population suffered from high prevalence and rapid development of caries decay (Wang et al., 2002; Phipps et al., 2012), making them a clinically significant age group and good candidates for longitudinal studies on the natural progression of dental caries and its associated microbiome shifts. This age group is also considered as having a “relatively stable primary dentition” in Pediatric dentistry. This means during this time, the primary dentition is already established and remains stable. After 5 years old, some permanent teeth may erupt and the oral cavity will change to mixed dentition. In our study, the average dt index of caries children increased rapidly from 2.0- at 6-month follow-up to 5.4 at 12-month follow-up, indicating these children suffered from severe caries decay, and their dental health would deteriorate if left untreated. Supra-gingival dental plaques were collected for microbial analysis due to their significantly higher bacterial diversity than that of saliva or the buccal mucosa (Xu et al., 2015) in healthy population. Meanwhile, considering that tooth surfaces are the places where caries occur, the investigation of microbial structural changes in supragingival plaque during caries initiation and development would be crucial for better understanding the etiology of dental caries (Hao et al., 2015). Since we conducted the study in confined kindergartens, all the children shared the same menu and the same oral cleaning habits during the daytime. However, their diet and oral hygiene habits at home would also be factors influencing these children’s oral microbiome. Based on the results from questionnaires survey, children included in the final CA and CF groups didn’t show significant difference in the frequency of eating sweets, frequency of eating sweets before sleep, drinking sugar-containing beverage or frequency of teeth brushing. In addition, oral health instructions were given to all the children and their parents during each follow-up to help keeping these children staying on a healthy diet and oral habit as much as possible. Considering all these factors, although there would be variations in diet and oral hygiene habits during their staying at home, we still consider this longitudinal study well-controlled.

Our longitudinal study revealed that the supragingival microbial community in CA and CF groups differed not only in the taxa composition, but also in their co-occurrence patterns (**Figure 2**). The significantly more extensive and strong correlations, both positive and negative, among bacterial species as well as the formation of tight bacterial clusters in CA group suggested more intense and specific interspecies interactions among supragingival microbiome. Specific microbial co-occurrence patterns have also been observed in supra-gingival microbiome in adult dental caries patients, suggesting coordinated bacterial interactions might contribute to caries development (Xiao et al., 2016). Like in most natural environments, bacterial species within oral microbiome form a complex system of interspecies interactions. These dynamic interactions are subjected to constant host modulation and other environmental perturbations and are the main driving force in shaping the structure and ultimately determine the function and ecology of microbial community (Baker et al., 2017). The co-occurrence of certain microbial taxa, particularly the presence of specific microbial complexes, can be considered as a driver for community composition and can be used as indicators for the presence of diseases (Jakubovics, 2015; Gomez and Nelson, 2017); such as “red” and “orange” complex has long been used as an indicator for the presence of periodontitis, another oral polymicrobial disease resulted from dysbiosis of subgingival microbial community (Socransky et al., 1998). Increased strength of microbial co-occurrence has been detected in the subgingival microbiome of periodontitis, while in the healthy states, the microbial species are less correlated in relative abundance and can vary significantly among different subjects (Shi et al., 2015). Furthermore, in periodontitis subjects, both disease- and health-associated species co-occur more often within community than in the disease resolved states in the same subjects (Shi et al., 2015). Our data were in line with these findings, suggesting coordinated microbial interactions could be important to the development of oral diseases, including both periodontitis and dental caries.

The knowledge of the oral microbiome development during early childhood is limited. A few studies focused on the microbial population of the oral cavity following birth (Gomez and Nelson, 2017). Some longitudinal studies followed and compared the oral microbiome of the same subjects after a relatively long period of time (Dzidic et al., 2018; Xu et al., 2018). Dzidic et al. (2018) recorded the oral microbiome development in 90 children, by collecting saliva at four time points before 2 years old and then at 7 years old. However, this study mainly recorded the development/changes at two stages, first was during primary dentition establishment and the other was after some permanent teeth have erupted. Since it has been documented that the eruption of permanent teeth would influence the oral microbiome (Shi et al., 2016, 2018), it should also be counted as a contributing factor in microbiome variation other than aging. Recently, Xu et al. (2018) reported an age-related variations in salivary microbial community of 4- to 6-year-old children. However, it was not mentioned how many children have permanent teeth emerged into the oral cavity at 6 years old. In our study, no obvious shifting patterns of the supragingival microbiome were observed in healthy children during the 12-month long observation. This could be partly due to the relatively short observation time. Another reason might be that the age we chose was at the beginning of the relative stable primary dentition period when the impact of eruption of permanent teeth on the microbiome is non-existent.

Our study revealed more drastic dynamic shift in co-occurrence pattern in the CA group during caries initiation and progression. The formation of tightly formed bacterial clusters during caries development suggested that these species might play important roles in caries formation by creating micro-ecological niches that favors the growth of caries-associated species. Among the OTUs that possessed the most connections with other OTUs in the CA group at 12-month follow-up, *Dolosigranulum* (OTU-179) is a member of the Carnobacteriaceae family, which are gram-positive lactic acid bacteria (**Figure 2**). Little is known about *Dolosigranulum*. The first genome of *Dolosigranulum* spp. was mapped by whole genome sequencing (*Dolosigranulum pigrum ATCC 51524, Broad Institute of Harvard and MIT*)^1^ not long ago (Biesbroek et al., 2014; Salter et al., 2017; Wang et al., 2018). In a recent 2-year longitudinal study to investigate dynamic alterations related to dental caries in salivary microbiota of preschool children with deciduous dentition, *Dolosigranulum* was found to be enriched in healthy group compared to caries group at 6-month follow-up, which is accordance with our result (Xu et al., 2018). Not much is known about the relationships of the four Gammaproteobacteria [*Pseudomonas* (OTU-170), *Acinetobacter* (OTU-207), Enterobacteriaceae (OTU-85), and Cardiobacteriaceae (OTU-57)] and their roles in caries development. However, one advantage of the sequencing analysis was its superiority in detecting new species (Xu et al., 2014). Further research should focus on these clusters from both CF and CA group at different time points, to investigate their interactions, their ecological roles as well as their impact on cariopathogenesis.

Intriguingly, the correlation networks revealed a remarkable increase of positive correlations among the CA-enriched species, as well as negative correlations between CA-enriched and CF-enriched species in the CA group, compared to the CF group (**Figure 4**). This finding suggested that during caries progression, the enhanced synergy among caries-associated bacteria and antagonistic interaction between CA-associated and CF-associated species might contribute to caries development (**Figure 4**). The large and tight bacterial clustering as well as the increased number and intensity of microbial correlations in CA group could result in limited while characteristic microbial structure patterns, which was consistent with our PCoA analysis of microbiome from the CA group (**Figure 1**).

Increasing lines of evidence showed that the pattern of relative abundance of metabolic modules/pathways could be indicative of healthy or certain diseased condition (Rudney et al., 2015; Xiao et al., 2016). Our preliminary microbiome metabolic pathways analysis data are in line with this notion showing increased abundance of pathways for carbohydrate metabolism in the CA group (**Figure 7**). Dental caries is the result of dissolution of the teeth by acid produced by carbohydrate-fermenting oral bacteria. Thus, the increased carbohydrate utilization capability could be a signature of caries-associated microbiome, which plays a significant role in caries initiation and development (Sheiham and James, 2015; Galvao-Moreira, 2016; James, 2016). However, the PICRUSt predictions has its limitations, it only includes 16S marker gene sequences which are corresponding to bacterial and archaeal genomes, and its ability to detect patterns also depends on the input data used (Langille et al., 2013). So, further studies are needed to focus on the key bacteria and functional genes that are involved in the carbohydrate metabolisms to explore their functions in the micro-ecological environment and impacts on promoting caries-associated microbial ecology.

Previous researches demonstrated decreased bacterial richness after caries onset (Tao et al., 2013). By applying PCR-DGGE analysis to detect changes in the microbial richness of dental plaque during caries development, Hao et al. (2015), reported a significant decrease in microbial richness of supragingival microbiome at 6 months, but not as early as 12 months, before caries onset. Our data are consistent with their finding, revealing remarkable differences in the supragingival plaque microbiome composition as well as correlations among bacteria between the CF and CA group even at the baseline (**Figures 1–4**). The current concept of polymicrobial diseases emphasize on the crucial role of microbiome in the pathogenesis/etiology of human microbial diseases such as caries, periodontitis, and colitis. Increasing lines of evidence indicated that microbial shift often precedes the disease onset (Yassour et al., 2016). Our data collaborated with these findings, suggested that long before the clinical manifestation of dental caries can be detected, the supragingival plaque microbiome compositions might have already undergone significant change.

The caries onset prediction model was constructed on the basis of the differential functional profile of the microbiomes between CA and CF group. Preliminary validation was done using the sample microbiome information from another subgroup (CF-6m subgroup), avoiding the possible information overlap with the subgroups (CA-0m and CF-0m subgroups) that were used for model construction. Further validation should be done using clinical samples in the future. Nevertheless, our study corroborated previous finding (Teng et al., 2015), demonstrated potential applications of the supragingival microbiome in caries prediction. It could give rise to future development of useful early diagnostic and prognostic tools that enable more specific, individualized therapies.

There were several limitations of this study. One of the main goals of this study was to investigate the dynamic shift in microbial composition during dental caries initiation and progression. So when designing the study, we excluded children who received dental treatment during the 6- and 12-month follow-up, so as to be certain that microbial changes during the disease progression can be captured/recorded without any intervention (such as filling). However, this exclusion obviously reduced the study’s power. It is our intention to design the experiment more effectively in future studies.

In all, recent advances in molecular biology have facilitated the profiling of the caries-related oral microbiome (Yang et al., 2012), however, the respect function and roles of these caries-associated species in disease onset and development are still poorly understood (Takahashi, 2015). This study illustrated the shift of supragingival microbiome during caries initiation and progression. Significant microbial shifts occur not only during caries development, but even in the sub-clinical state. One caries-onset prediction model was constructed based on the supragingival microbiome profiles. Our data suggested coordinated microbial interactions could be important to caries pathogenesis, and the microbial shifts prior to the onset of caries might potentially be used for the early diagnosis and intervention of caries. Future work should focus on revealing the intricate interspecies interaction network through multi-omics analysis and studying the underlying mechanisms driving the transition from eubiotic to dysbiotic state.

## Author Contributions

HX wrote the paper. JT analyzed the sequence data. WH collected the samples. QZha, QZho, and WS performed DNA extraction and other lab methods. MQ conceived the work, designed the experiments, and critically revised the manuscript. XH and FC designed the experiments and critically revised the manuscript.

## Conflict of Interest Statement

The authors declare that the research was conducted in the absence of any commercial or financial relationships that could be construed as a potential conflict of interest.

## Footnotes

1 http://www.broadinstitute.org/

## Supplementary Material

FIGURE S1 Flow chart of inclusion and exclusion criteria.

FIGURE S2 Microbial compositions of all samples at phylum level. **(a–c)** Each column represented the relative abundance of microbial components in a single sample. Sequence annotation was performed with the aid of the Ribosomal Database Project (RDP).

FIGURE S3 Rarefaction curves obtained using RDP database with 97% similarity. **(a,b)** Rarefaction curves of Chao 1 and observed species indexes. **(c,d)** Rarefaction curves of Shannon and Simpson indexes.

FIGURE S4 Alpha diversity. **(a,b)** Chao 1 and Observed OTUs indexes exhibited bacterial community richness. **(c,d)** Shannon and Simpson indexes exhibited bacterial community diversity.

FIGURE S5 Receiver operating characteristic (ROC) curve of the prediction model of caries onset.

TABLE S1 Comparison of oral behavioral habits between caries-free (CF) and caries-affected (CA) groups at baseline, six months follow-up and twelve months follow-up.

TABLE S2 General information of participants and diversity estimation at 3% dissimilarity from the pyrosequencing analysis.

TABLE S3 Differently distributed pathways between caries-free and caries-affected children.

TABLE S4 Prediction result of validation samples using caries-onset prediction model.

DATA SHEET S1 OTU annotation of all taxonomy.
