# Supplementary figures and images for: Oral Microbiome Shifts From Caries-Free to Caries-Affected Status in 3-Year-Old Chinese Children: A Longitudinal Study
Source: Front Microbiol. 2018 Aug 28;9:2009. doi: 10.3389/fmicb.2018.02009 (PMC6121080; doi:10.3389/fmicb.2018.02009)

## Inclusion

## Exclusion

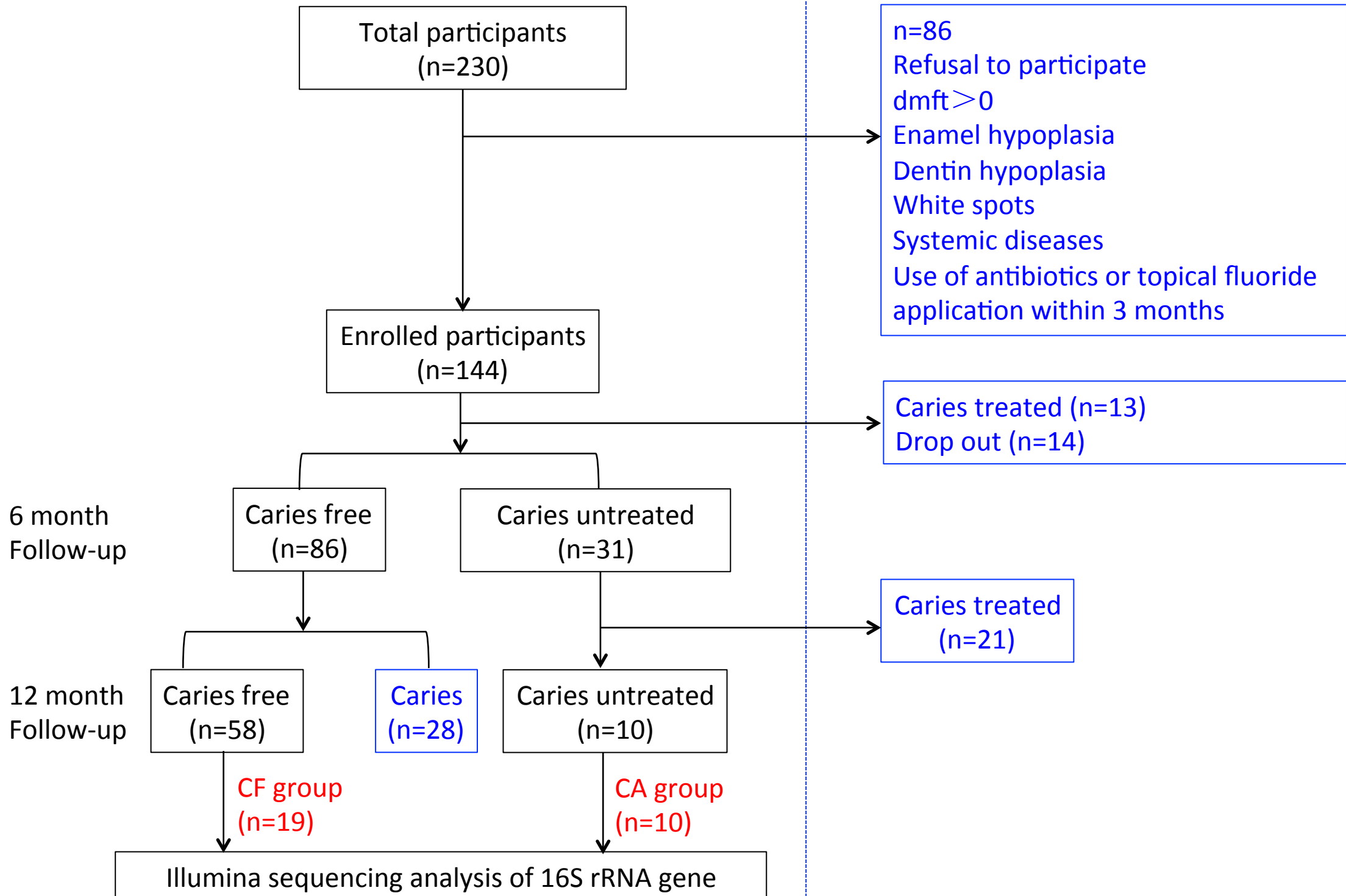

Supplement: FIGURE S1 — Flow chart of inclusion and exclusion criteria. [file Data_Sheet_2.PDF]

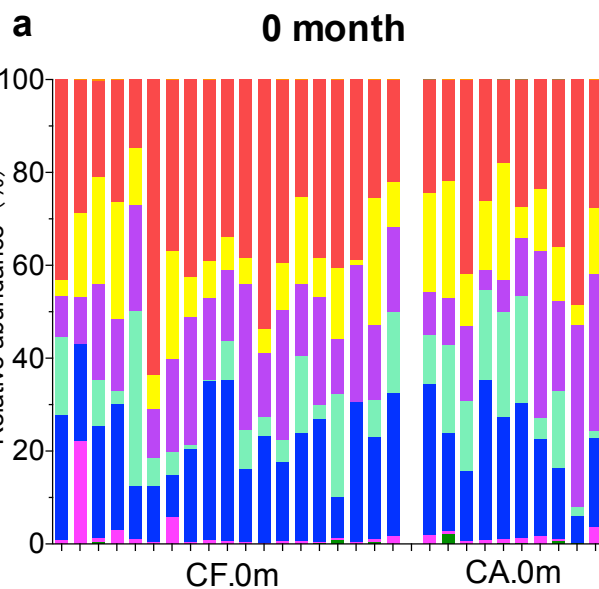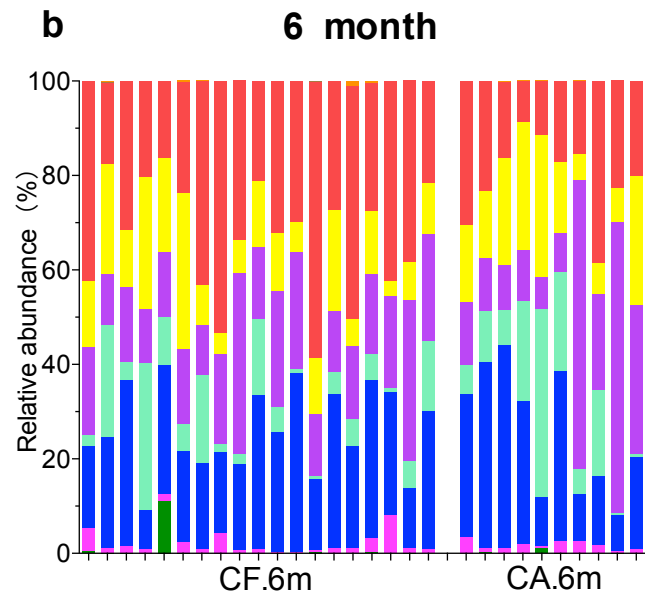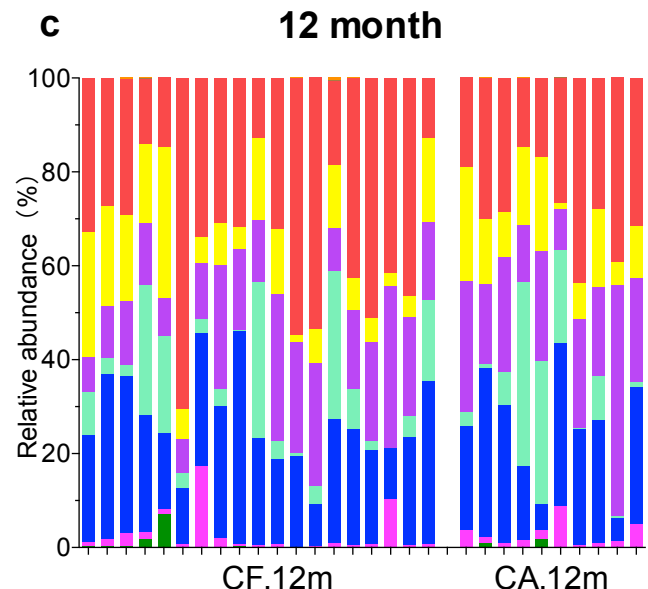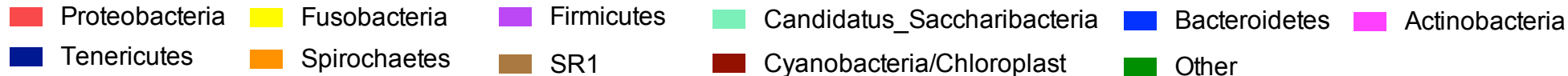

Supplement: FIGURE S2 — Microbial compositions of all samples at phylum level. (a–c) Each column represented the relative abundance of microbial components in a single sample. Sequence annotation was performed with the aid of the Ribosomal Database Project (RDP). [file Data_Sheet_3.PDF]

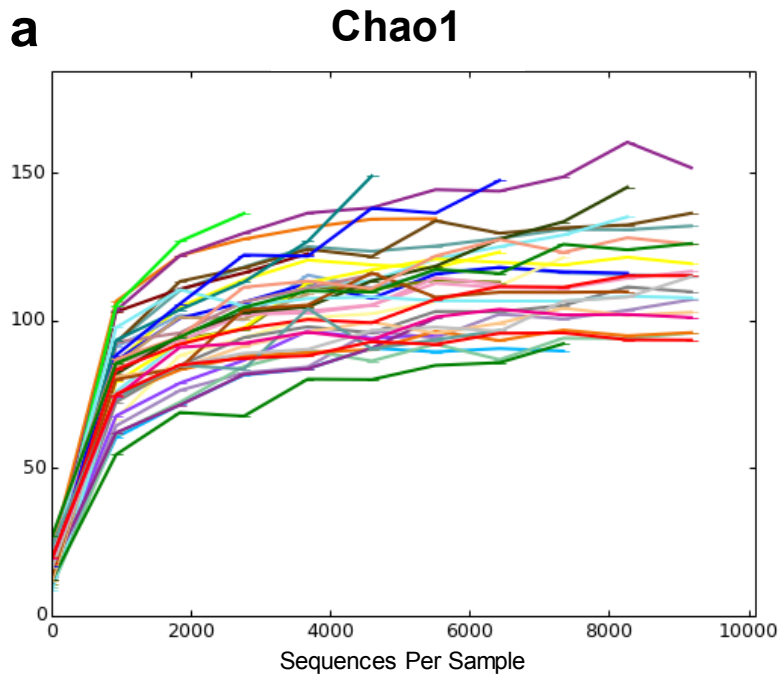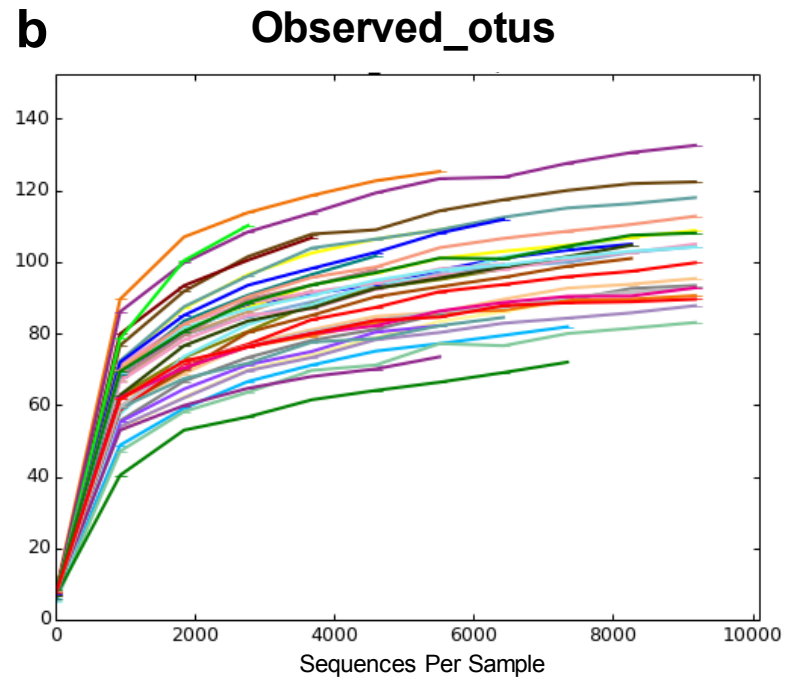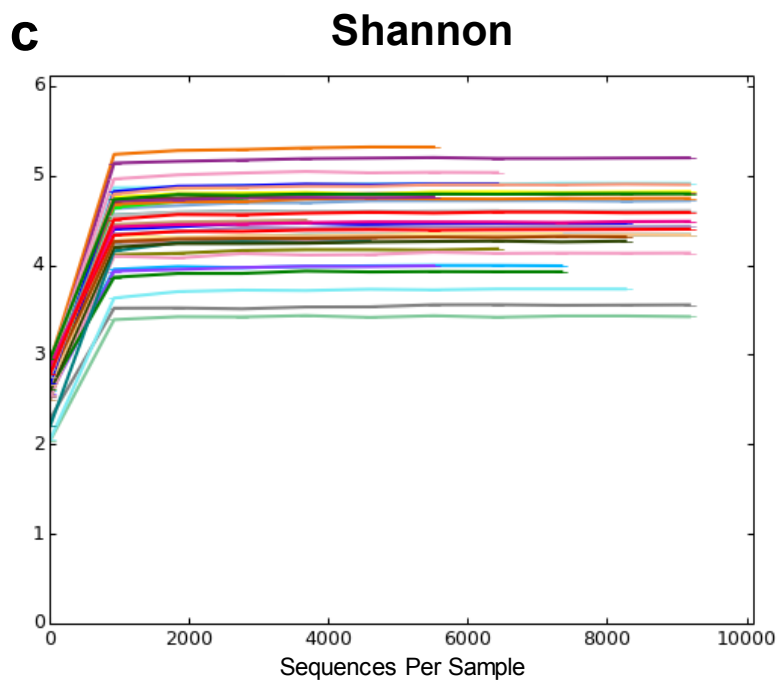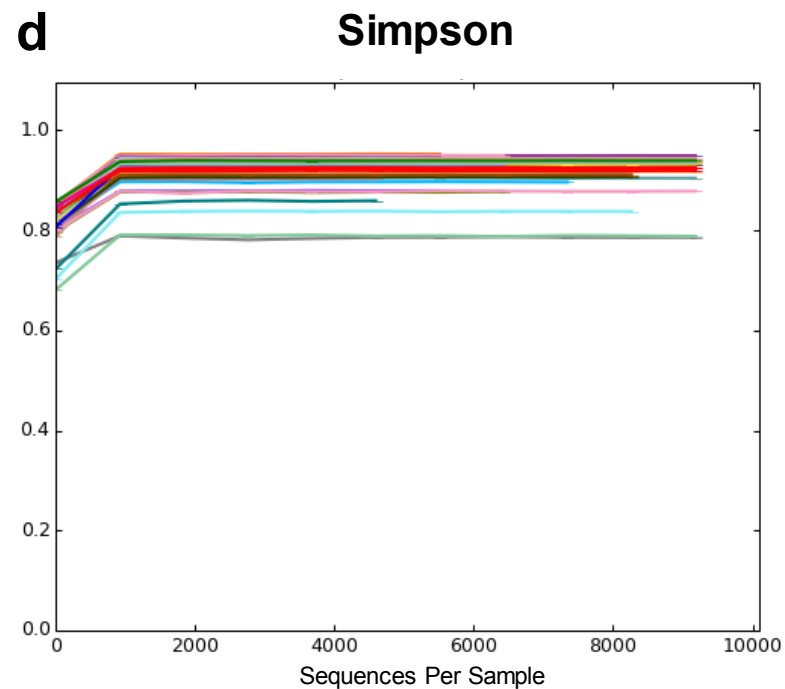

Supplement: FIGURE S3 — Rarefaction curves obtained using RDP database with 97% similarity. (a,b) Rarefaction curves of Chao 1 and observed species indexes. (c,d) Rarefaction curves of Shannon and Simpson indexes. [file Data_Sheet_4.PDF]

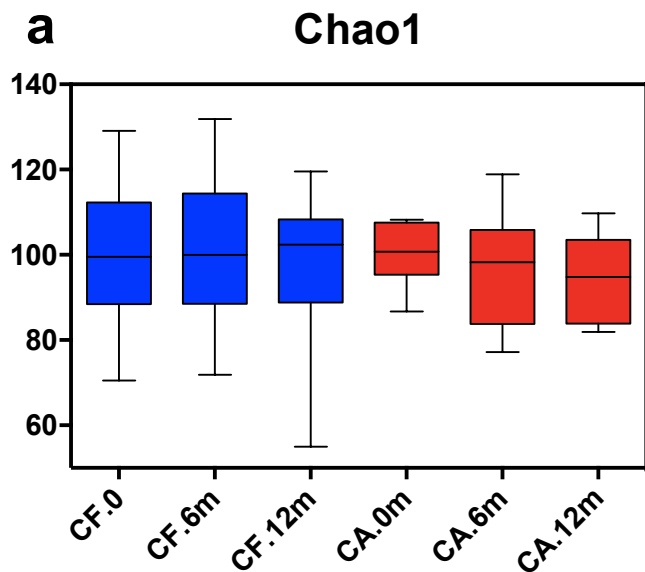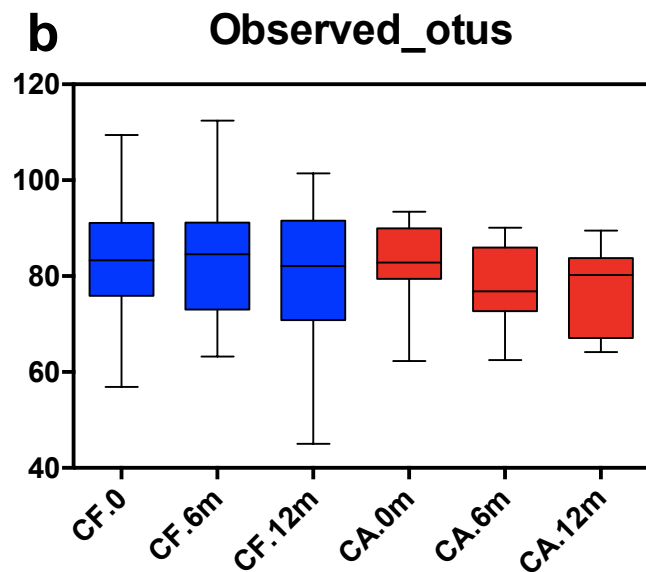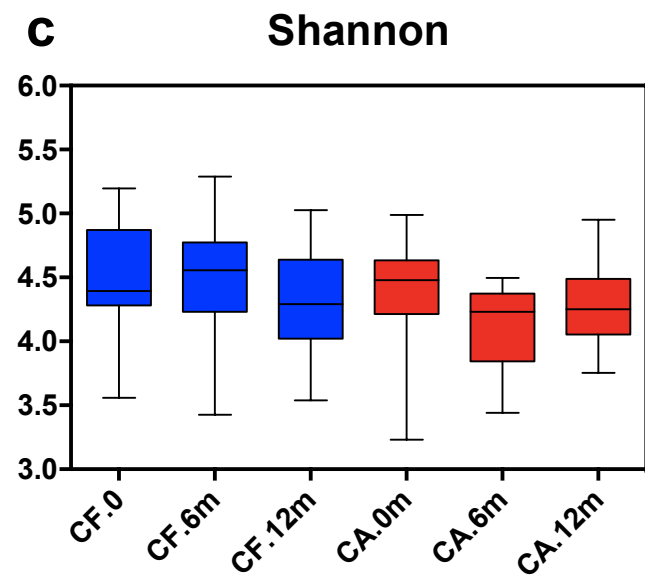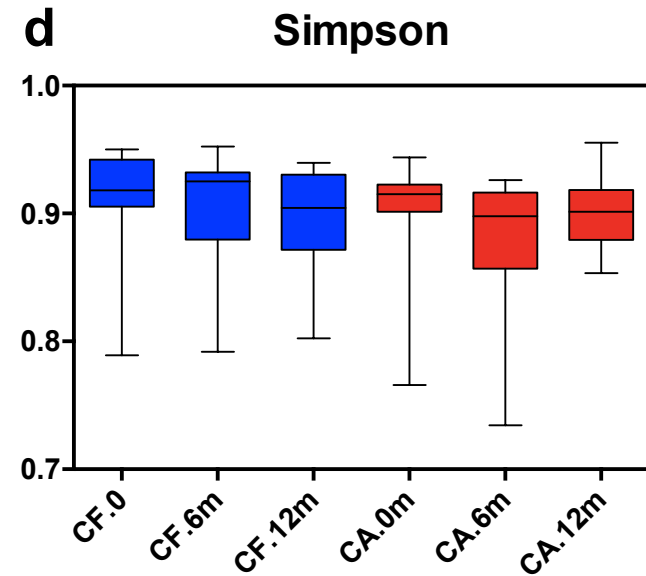

Supplement: FIGURE S4 — Alpha diversity. (a,b) Chao 1 and Observed OTUs indexes exhibited bacterial community richness. (c,d) Shannon and Simpson indexes exhibited bacterial community diversity. [file Data_Sheet_5.PDF]

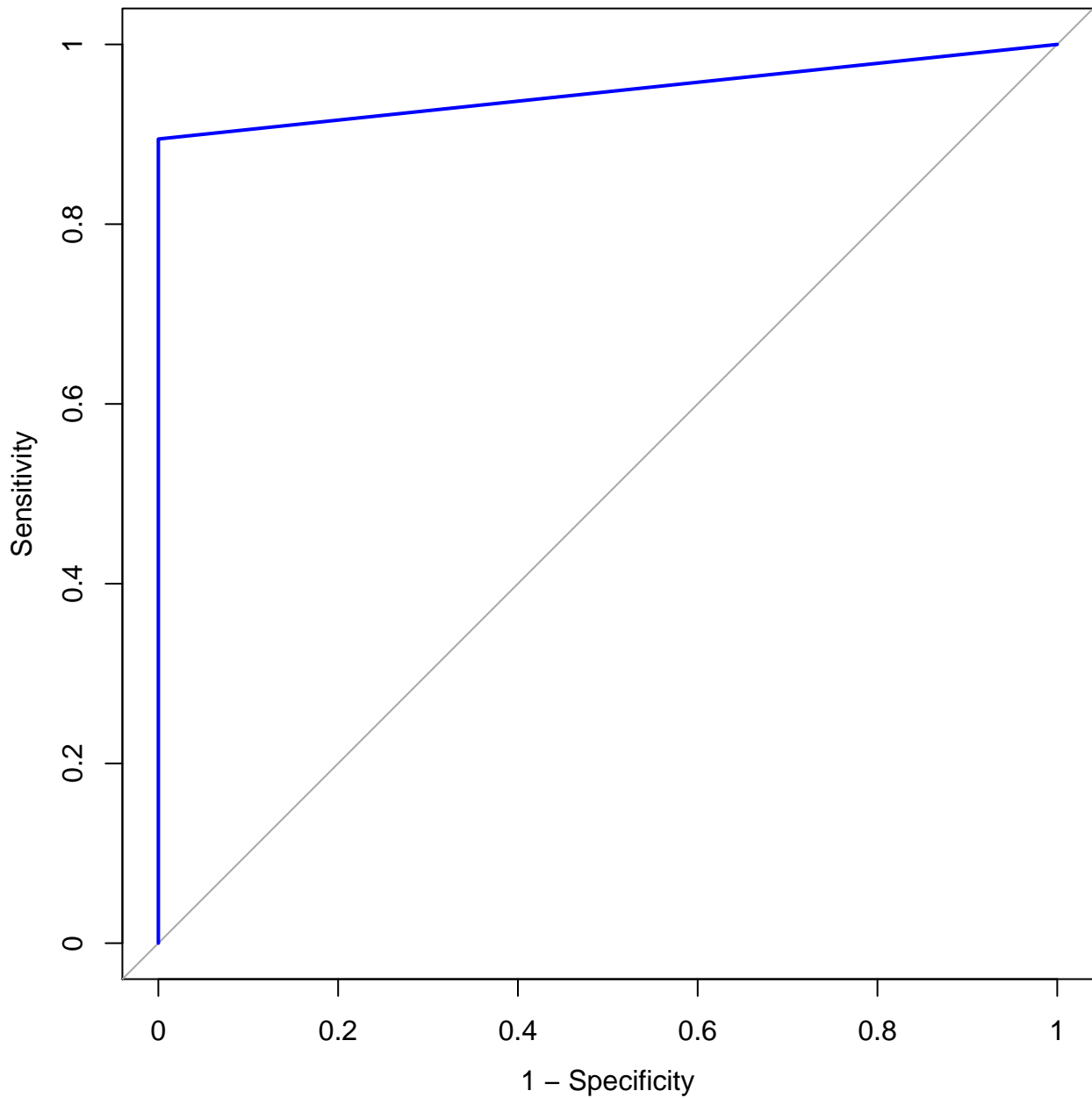

Supplement: FIGURE S5 — Receiver operating characteristic (ROC) curve of the prediction model of caries onset. [file Data_Sheet_6.PDF]
