# Supplementary material for: Oral Microbiome Shifts From Caries-Free to Caries-Affected Status in 3-Year-Old Chinese Children: A Longitudinal Study
Source: Front Microbiol. 2018 Aug 28;9:2009. doi: 10.3389/fmicb.2018.02009 (PMC6121080; doi:10.3389/fmicb.2018.02009)
Supplement: TABLE S1 — Comparison of oral behavioral habits between caries-free (CF) and caries-affected (CA) groups at baseline, six months follow-up and twelve months follow-up. [file Table_1.DOCX]

Table S1. Comparison of oral behavioral habits between caries-free（CF）and caries-affected (CA) groups at baseline, six months follow-up and twelve months follow-up.

| Variable ^a^ | Baseline | | Six months review (%) | | Twelve months review (%) | |
| --- | --- | --- | --- | --- | --- | --- |
|  | CF group | CA  group | CF group | CA group | CF group | CA group |
| Frequency of eating sweets |  |  |  |  |  |  |
| ≥3 time/day | 2 | 0 | 0 | 0 | 0 | 0 |
| <3 time/day | 14 | 9 | 19 | 10 | 19 | 10 |
| Frequency of eating sweets before sleeping |  |  |  |  |  |  |
| ≥3 time/week | 2 | 0 | 0 | 1 | 1 | 1 |
| <3 time/week | 15 | 10 | 19 | 7 | 18 | 9 |
| Frequency of tooth brushing |  |  |  |  |  |  |
| ≥1 time/day | 15 | 9 | 15 | 7 | 15 | 9 |
| <1 time/day | 3 | 1 | 4 | 2 | 4 | 1 |
| Drink |  |  |  |  |  |  |
| Only or mainly water, sugar-containing beverage occasionally | 14 | 9 | 19 | 10 | 11 | 6 |
| Mainly sugar-containing beverage, water occasionally | 2 | 0 | 0 | 0 | 0 | 0 |

^a^ By Fisher’s exact test for each variable; all comparisons not significant (p > 0.05).
