# Supplementary material for: Oral Microbiome Shifts From Caries-Free to Caries-Affected Status in 3-Year-Old Chinese Children: A Longitudinal Study
Source: Front Microbiol. 2018 Aug 28;9:2009. doi: 10.3389/fmicb.2018.02009 (PMC6121080; doi:10.3389/fmicb.2018.02009)
Supplement: TABLE S2 — General information of participants and diversity estimation at 3% dissimilarity from the pyrosequencing analysis. [file Table_2.DOCX]

Table S2. General information of participants and diversity estimation at 3 % dissimilarity from the pyrosequencing analysis

| Sample | Gender | Age^a^ (month) | dt | | | Final reads | | | OTU^b^ num | | |
| --- | --- | --- | --- | --- | --- | --- | --- | --- | --- | --- | --- |
|  |  |  | 0 month | 6 month | 12 month | 0 month | 6 month | 12 month | 0 month | 6 month | 12 month |
| CA-01 | Female | 36 | 0 | 1 | 4 | 9220 | 9990 | 8990 | 109 | 84 | 92 |
| CA-02 | Female | 37 | 0 | 4 | 9 | 13315 | 11520 | 13377 | 106 | 94 | 108 |
| CA-03 | Male | 40 | 0 | 3 | 8 | 9184 | 9839 | 13714 | 80 | 94 | 94 |
| CA-04 | Male | 41 | 0 | 2 | 10 | 9075 | 10877 | 20791 | 101 | 98 | 109 |
| CA-05 | Female | 41 | 0 | 2 | 3 | 6462 | 12224 | 9975 | 108 | 132 | 110 |
| CA-06 | Male | 39 | 0 | 3 | 5 | 5208 | 12487 | 4976 | 101 | 111 | 79 |
| CA-07 | Male | 39 | 0 | 1 | 7 | 9534 | 13584 | 13144 | 111 | 109 | 106 |
| CA-08 | Male | 39 | 0 | 2 | 2 | 11106 | 9077 | 9692 | 123 | 111 | 113 |
| CA-09 | Female | 38 | 0 | 1 | 5 | 5643 | 6341 | 5250 | 93 | 88 | 74 |
| CA-10 | Male | 36 | 0 | 1 | 1 | 3808 | 8226 | 9355 | 86 | 109 | 106 |
| CF-01 | Male | 38 | 0 | 0 | 0 | 10410 | 7218 | 4990 | 102 | 115 | 100 |
| CF-02 | Male | 38 | 0 | 0 | 0 | 9448 | 13490 | 16559 | 91 | 115 | 125 |
| CF-03 | Female | 41 | 0 | 0 | 0 | 9435 | 12242 | 7029 | 133 | 113 | 111 |
| CF-04 | Male | 39 | 0 | 0 | 0 | 10427 | 12214 | 9499 | 105 | 109 | 122 |
| CF-05 | Female | 37 | 0 | 0 | 0 | 19826 | 14354 | 9583 | 132 | 129 | 114 |
| CF-06 | Male | 41 | 0 | 0 | 0 | 10376 | 3494 | 6166 | 96 | 117 | 96 |
| CF-07 | Male | 41 | 0 | 0 | 0 | 13822 | 11726 | 13677 | 119 | 108 | 90 |
| CF-08 | Female | 38 | 0 | 0 | 0 | 9747 | 11834 | 7323 | 96 | 87 | 89 |
| CF-09 | Male | 41 | 0 | 0 | 0 | 10047 | 7592 | 10505 | 89 | 90 | 83 |
| CF-10 | Male | 41 | 0 | 0 | 0 | 9257 | 26188 | 5580 | 93 | 109 | 115 |
| CF-11 | Female | 37 | 0 | 0 | 0 | 7758 | 3977 | 4486 | 82 | 93 | 91 |
| CF-12 | Male | 36 | 0 | 0 | 0 | 7324 | 5804 | 6186 | 101 | 83 | 77 |
| CF-13 | Male | 41 | 0 | 0 | 0 | 4613 | 5252 | 5609 | 98 | 105 | 95 |
| CF-14 | Female | 39 | 0 | 0 | 0 | 3747 | 8636 | 6309 | 107 | 105 | 116 |
| CF-15 | Male | 40 | 0 | 0 | 0 | 8933 | 11122 | 11646 | 102 | 92 | 119 |
| CF-16 | Male | 41 | 0 | 0 | 0 | 8736 | 5911 | 9214 | 106 | 127 | 106 |
| CF-17 | Female | 38 | 0 | 0 | 0 | 7416 | 6348 | 5169 | 72 | 75 | 86 |
| CF-18 | Male | 39 | 0 | 0 | 0 | 6947 | 8314 | 7670 | 113 | 107 | 88 |
| CF-19 | Male | 40 | 0 | 0 | 0 | 7000 | 6827 | 2773 | 101 | 86 | 45 |

^a^ Age: age at enrollment.

^b^ The operational taxonomic units (OTUs) were defined with 3 % dissimilarity level.
