# Supplementary material for: Oral Microbiome Shifts From Caries-Free to Caries-Affected Status in 3-Year-Old Chinese Children: A Longitudinal Study
Source: Front Microbiol. 2018 Aug 28;9:2009. doi: 10.3389/fmicb.2018.02009 (PMC6121080; doi:10.3389/fmicb.2018.02009)
Supplement: TABLE S3 — Differently distributed pathways between caries-free and caries-affected children. [file Table_3.DOCX]

Table S3. Differently distributed pathways between caries-free and caries-affected children.

|  | CF group | CA group |
| --- | --- | --- |
| Baseline | Glyoxylate and dicarboxylate metabolism | Protein export |
|  | Aminobenzoate degradation | DNA repair and recombination proteins |
|  | Benzoate degradation | DNA replication |
|  |  | DNA replication proteins |
|  |  | Homologous recombination |
|  |  | Mismatch repair |
|  |  | Transcription factors |
|  |  | Aminoacyl-tRNA biosynthesis |
|  |  | Ribosome |
|  |  | Amino acid related enzymes |
|  |  | Cysteine and methionine metabolism |
|  |  | Lysine biosynthesis |
|  |  | Peptidoglycan biosynthesis |
|  |  | Pantothenate and CoA biosynthesis |
|  |  | Translation proteins |
| 6 month | Bacterial motility proteins | Protein export |
|  | Secretion system | DNA replication |
|  | Phenylalanine metabolism | DNA replication proteins |
|  | Tryptophan metabolism | Mismatch repair |
|  | Valine, leucine and isoleucine degradation | Nucleotide excision repair |
|  | Glyoxylate and dicarboxylate metabolism | Aminoacyl-tRNA biosynthesis |
|  | Biosynthesis of unsaturated fatty acids | Ribosome |
|  | Fatty acid metabolism | Cysteine and methionine metabolism |
|  | Lipid biosynthesis proteins | Amino sugar and nucleotide sugar metabolism |
|  | Synthesis and degradation of ketone bodies | Fructose and mannose metabolism |
|  | Glutathione metabolism | Glycolysis / Gluconeogenesis |
|  | beta-Alanine metabolism | Starch and sucrose metabolism |
|  | Geraniol degradation | Carbon fixation in photosynthetic organisms |
|  | Aminobenzoate degradation | Photosynthesis |
|  | Benzoate degradation | Photosynthesis proteins |
|  | Drug metabolism - cytochrome P450 | Glycosyltransferases |
|  | Metabolism of xenobiotics by cytochrome P450 | Peptidoglycan biosynthesis |
|  | Naphthalene degradation | Steroid biosynthesis |
|  | Glycan biosynthesis and metabolism | Selenocompound metabolism |
|  |  | Purine metabolism |
|  |  | Pyrimidine metabolism |
| 12month | Epithelial cell signaling in Helicobacter pylori infection | Ascorbate and aldarate metabolism |
|  |  | Pentose and glucuronate interconversions |
