# Supplementary material for: Oral Microbiome Shifts From Caries-Free to Caries-Affected Status in 3-Year-Old Chinese Children: A Longitudinal Study
Source: Front Microbiol. 2018 Aug 28;9:2009. doi: 10.3389/fmicb.2018.02009 (PMC6121080; doi:10.3389/fmicb.2018.02009)
Supplement: TABLE S4 — Prediction result of validation samples using caries-onset prediction model. [file Table_4.DOCX]

Table S4. Prediction result of validation samples using caries-onset prediction model.

| CF.6m  samples | Probability of caries-affected at 12 month | Probability of caries-free at 12 month | Result |
| --- | --- | --- | --- |
| 1 | 0.13428571 | 0.8657143 | right |
| 2 | 0.27428571 | 0.7257143 | right |
| 3 | 0.16285714 | 0.8371429 | right |
| 4 | 0.49285714 | 0.5071429 | right |
| 5 | 0.31000000 | 0.6900000 | right |
| 6 | 0.04714286 | 0.9528571 | right |
| 7 | 0.12142857 | 0.8785714 | right |
| 8 | 0.38571429 | 0.6142857 | right |
| 9 | 0.28428571 | 0.7157143 | right |
| 10 | 0.19428571 | 0.8057143 | right |
| 11 | 0.28428571 | 0.7157143 | right |
| 12 | 0.19285714 | 0.8071429 | right |
| 13 | 0.13428571 | 0.8657143 | right |
| 14 | 0.35428571 | 0.6457143 | right |
| 15 | 0.36285714 | 0.6371429 | right |
| 16 | 0.13857143 | 0.8614286 | right |
| 17 | 0.16000000 | 0.8400000 | right |
| 18 | 0.55142857 | 0.4485714 | wrong |
| 19 | 0.71428571 | 0.2857143 | wrong |
